# Suum Cuique

**Published:** 1853-12

**Authors:** 


SUUM CUIQUE.—Our editorial friends in Chicago should exercise a benevolent prudence, as to the particular direction in which they throw their dangerous missiles. In copying and commenting upon a characteristic paragraph from a "Keokuk Medical Journal," they would have shielded the innocent, and protected us from an imputation, had they specified the particular "Keokuk Journal" alluded to. The back numbers of the Iowa Medical Journal do not contain the paragraph in question; but a Medical Journal has been published in this city, in which we presume the article must have originated. It becomes necessary for us to explain that we have no association with the proprietor of that publication, and that he has no connection whatever with this Journal, or with the Medical Institution in this city. We are certainly, therefore, not disposed to father the paragraph referred to. So, dear Dr. North-West, do please remove from us that stigma, for

"Thou can'st not say that I did it."

This explanation is also necessary on account of a misapprehension of the relative position of our Journal, by two or three other Medical Journals in different parts of the country.

